# Multidisciplinary Role of Microfluidics for Biomedical and Diagnostic Applications: Biomedical Microfluidic Devices

**DOI:** 10.3390/mi8120343

**Published:** 2017-11-27

**Authors:** Kwang W. Oh

**Affiliations:** SMALL (Sensors and MicroActuators Learning Lab), Department of Electrical Engineering & Department of Biomedical Engineering, University at Buffalo, State University of New York (SUNY-Buffalo), Buffalo, NY 14260, USA; kwangoh@buffalo.edu; Tel.: +1-716-645-1025

Life scientists are closely working with engineers to solve biological and biomedical problems through the application of engineering tools. For engineers involved in this collaborative work, new knowledge is created in their own disciplines. For example, the science and technology at the interface of biomedical sciences and microfluidics has played a significant role in ushering in recent advances in genomics, proteomics, single cell analysis, and lab-on-chip (LOC)/point-of-care (POC) diagnostics. Moreover, this interplay has led to the miniaturization of biomedical microfluidic devices to replace routine analyses and diagnostics, featuring a high degree of system integration; improved potential for automation, control, and high-throughput processing; small volumes of samples and reagents; reduced cost; greater reliability and sensitivity; personalization and disposability; and shorter bioassay times.

This special issue of *Micromachines* entitled ‘Biomedical Microfluidic Devices’ provides a discussion of the technical challenges associated with developing microfluidic devices for biomedical and diagnostic applications. Addressing these challenges requires technological advances in many areas, including sensors [1,2], actuators [3], materials [4,5], microfabrication techniques [6], simulations and models [7,8,9], and platform technologies [10,11,12]. This special issue consists of 12 high-quality papers, including two insightful review articles [4,12].

*Sensors*: The integration of sensors in microfluidic devices has great potential in stand-alone or hand-held systems for various biological and biomedical applications. Using an electroceutical approach in a simple microfluidic device, Berthelot et al. [1] report the impact of varying electrical currents and acetic acid concentrations on bacterial motility dynamics. Khashayer et al. [2] developed an electrochemical sensor integrated with a microfluidic cartridge to study serum levels of different bone markers for the potential applicability of osteoporosis care.

*Actuators*: Successful commercialization of LOC/POC devices has been hindered owing to the lack of reliable microfluidic actuators, such as microvalves and micropumps. To overcome this challenge, Kinahan et al. [3] present a chemically actuated valving mechanism through gas release from baking powder that is initially dry-stored on a centrifugally driven biomedical microfluidic device.

*Materials*: A review article by Ma et al. [4] summarizes the multidisciplinary role of microfluidics for biomaterials in areas ranging from synthesis technologies to biological applications. The authors highlight the superior properties and performance of functional biomaterials synthesized by microfluidics, which arise because their morphology and composition can be controlled through unique microfluidic scaling effects, such as laminar streaming flow, high surface-to-volume ratio, and improved heat and mass transfer. They categorize microfluidic-based biomaterials into four groups according to the material dimensionality: 0D for particulate materials, 1D for fibrous materials, 2D for sheet materials, and 3D for construct forms of materials. In particular, they highlight the microfluidic synthesis technologies for 0D particulate and 1D fibrous biomaterials, and focus on their biomedical applications. In a related original research article, Higashi et al. [5] report the synthesis of hollow hydrogel microfibers containing microorganisms for mass-cultivation in an open system by using a co-flowing microfluidic device.

*Microfabrication techniques*: It is important to develop simple, low-cost fabrication methods for biomedical microfluidic devices. In particular, PDMS (polydimethylsiloxane) is considered to be a good material for many biomedical microfluidic devices, because of its rapid prototyping capability using soft-lithography techniques as well as many advantageous properties, such as optical transparency, a non-toxic nature, and biocompatibility. However, many conventional soft-lithography techniques are unsuitable for the fabrication of non-conventional microfluidic structures and devices. Working to overcome this problem, Lee et al. [6] recently developed a simple fabrication method to form microwell array structures by transferring the patterns of a PDMS stamp onto a glass substrate. They used the microarrays to study cell-to-cell adhesion and smooth muscle differentiation using four different types of array patterns (i.e., rectangle, bowtie, wide-rhombus, and rhombus). They also suggest that the method could be used to fabricate thin glass—PDMS—glass biomedical microfluidic devices, by transferring microfluidic channel patterns on a glass substrate and sealing the channel with another glass substrate.

*Simulations and models*: The precise control of fluid flow in complex microfluidic structures and circuits represents another key challenge. A rigorous analysis and optimization of flow through such devices can be achieved using computational fluid dynamic (CFD) analysis. For example, Azzopardi et al. [7] improved the uniformity of flow across a large-area resonant biosensor by using COMSOL multiphysics. By using ANSYS Fluent, Li et al. [8] optimized microfluidic microfilters of circulating tumor cells to achieve higher throughput, less cellular damage, and better efficiency. In addition, Mizoue et al. [9] proposed an analytical model to achieve fast and accurate cell manipulation in a deformable PDMS-based microfluidic device, by studying its second-order transfer function of macro-to-micro manipulation (or input-to-output relationship).

*Platform technologies*: A well-defined microfluidic platform provides an efficient solution to implement a combination of various unit operations and unit processes. For example, Tsai’s group [10,11] developed a pressure-driven microfluidic constriction platform to evaluate on-chip red blood cell deformability. In general, according to a distinct set of fluidic manipulation processes, biomedical microfluidic devices can be categorized into different microfluidic platforms, for example, capillary-driven, pressure-driven [7,8,9,10,11], vacuum-driven, electrowetting-driven, centrifugal-driven [3], droplet-based [4], and paper-based microfluidic platforms, among others. An article by Basha et al. [12] provides a comprehensive review of the core processes implemented in POC devices (e.g., lysis techniques, nucleic acid extraction, amplification of specific DNA/RNA, and genomic identification methods) and microfluidic platforms suitable for molecular diagnosis (e.g., paper-based, centrifugal-based, and electrowetting-based microfluidic platforms).

I am sure that this special issue will be of high interest for life scientists and engineers working in the multidisciplinary field of biomedical microfluidic devices, as well as for readers of other areas of research in micro- and nanoscale science, devices, and applications. I look forward to you sharing your stories of progress in this exciting area in *Micromachines*!
